# Repeated analyses of national clinical audit reports demonstrate improvements in feedback methods

**DOI:** 10.1186/s43058-020-00089-3

**Published:** 2020-11-25

**Authors:** Tasneem Khan, Sarah Alderson, Jill J. Francis, Fabiana Lorencatto, John Grant-Casey, Simon J. Stanworth, Robbie Foy

**Affiliations:** 1 Leeds Institute for Health Sciences, University of Leeds, Leeds, UK; 2 School of Health Sciences, City University of London, London, UK; 3 Centre for Behaviour Change, University College London, London, UK; 4 NHS Blood and Transplant, Oxford, UK; 5 Transfusion Medicine, NHS Blood and Transplant, Oxford, UK; 6 Department of Haematology, Oxford University Hospitals NHS Foundation Trust, Oxford, UK; 7 Radcliffe Department of Medicine, University of Oxford, and NIHR Oxford Biomedical Research Centre, Oxford, UK

**Keywords:** Audit and feedback, Healthcare quality improvement, Implementation Science

## Abstract

**Background:**

There is growing interest in the impact of national clinical audit programmes on the quality of healthcare. There is also an evolving evidence-base for enhancing the design and delivery of audit and feedback. We assessed the extent to which a sample of UK national clinical audit feedback reports met a set of good practice criteria over three time points.

**Methods:**

We undertook three cross-sectional content analyses. We developed good practice criteria for the content and delivery of feedback based upon evidence, behavioural theory and expert opinion. We applied these to a feedback reports from 23 national audits listed on the Healthcare Quality Improvement Partnership (HQIP) website in November 2015. We repeated our assessments in January 2017 for 20 repeat feedback reports, after HQIP had published reporting guidance for national audits, and in August 2019 for a further 14 repeat feedback reports. We verified our assessments, where possible, with audit leads.

**Results:**

Feedback reports consistently included strengths at baseline, including past or planned repeated audit cycles (21; 91%), stating the importance of the topic in relation to patient care (22; 93%), using multi-modal data presentation (23; 100%), and summarising key findings (23; 100%).

We observed improvements over subsequent assessments, so that by 2019, at least 13 out of 14 (93%) feedback reports presented easily identifiable key findings and recommendations, linked recommendations to audit standards, and proposed easily identifiable action plans. Whilst the use of regional comparators did not improve, audit leads highlighted that programmes now provide local data via additional means.

The main shortcoming was the time lag between data collection and feedback; none of the 14 reports assessed in 2019 presented performance data less than 6 months old. Audit leads highlighted that some of these data might be available via programme websites.

**Conclusion:**

We identified increased adherence to good practice in feedback by national clinical audit programmes that may enhance their impact on service delivery and outcomes. There is scope for improvement, especially in the recency of performance data. With further refinements, a criterion-based assessment offers an efficient means of monitoring the quality of national clinical audit feedback reports.

**Supplementary Information:**

The online version contains supplementary material available at 10.1186/s43058-020-00089-3.

Contributions to literature

- National clinical audit programmes have a key role in driving improvements in population healthcare and health but the extent to which they employ best practice in audit and feedback is unknown.
- We used criteria based on current evidence, best practice and expert opinion to identify strengths and scope for improvement in feedback reports delivered by UK national clinic audit programmes.
- We demonstrated improvements over time but also significant scope for enhancing impact, including the need to reduce the time lag between data collection and feedback.

## Background

Audit and feedback aims to improve patient care by reviewing clinical performance against explicit standards. A Cochrane review of 140 randomised trials found that audit and feedback had modest effects on improving patient processes of care, leading to a median 4.3% absolute improvement (interquartile range 0.5 to 16%) in compliance with recommended practice [1]. One quarter of audit and feedback interventions had a relatively large, positive effect on quality of care, whilst another quarter had a negative or null effect. Meta-regression indicated larger effect sizes when baseline performance is low, feedback is delivered through a supervisor or colleague, feedback is provided more than once, feedback is delivered in both verbal and written formats and feedback includes both explicit targets and an action plan. There is scope to improve the effectiveness of audit and feedback in both practice and research [2].

The National Clinical Audit and Patient Outcomes Programme (NCAPOP) supports national clinical audits and confidential enquiries into patient outcomes commissioned by the Healthcare Quality Improvement Partnership (HQIP) on behalf of NHS England and devolved nations. These national clinical audit programmes address a variety of priorities across secondary care, such as stroke and cancer, and in primary care, such as diabetes. National clinical audit programmes aim to improve health outcomes by enabling those who commission, deliver and receive healthcare to measure and improve care delivery. An earlier self-assessment survey of 42 NCAPOP audits and enquiries largely focused on structures, governance and audit design but did not refer to the wider effectiveness evidence base on feedback when summarising how each programme disseminated findings [3]. A more recent analysis of feedback from one national audit programme identified a number of areas for improvement informed by evidence and theory, such as improving the behavioural specificity of audit standards, feedback and recommendations for change [4]. There is a growing recognition of the need for audit programmes to place as much emphasis on delivering effective feedback as on data collection if they are to achieve population impacts [5, 6].

We assessed the extent to which feedback and materials in national clinical audit reports reflect good practice recommendations based upon evidence and theory and described changes in report content over time.

## Methods

We undertook three cross-sectional content analyses of feedback reports from national clinical audits and their dissemination methods.

Our initial sample included feedback reports for all UK national clinical audits published on the HQIP website as of 1 November 2015. We identified the most recent feedback report from each audit programme. We then identified the latest feedback reports as of 31 January 2017 and 31 August 2019, respectively, reviewing only those that had repeat audits within this timeframe to allow direct comparison.

We analysed the contents of the feedback reports using a checklist created from Cochrane review recommendations [1], behaviour change theory relevant to audit and feedback [7], and published expert opinion [2]. Two reviewers (TK and RF) independently piloted a draft checklist with a sub-sample of three feedback reports and refined the checklist. Data from the pilot study using the refined checklist were included in the final dataset. One reviewer (TK) extracted data for all audits and coded for the presence or absence of criteria in the checklist. There were some cases where criteria were not applicable, such as lack of past performance data in the case of a first audit cycle, but we made the decision to apply all criteria to every feedback report to reduce subjectivity in our assessment.

We gained permission from HQIP to contact audit leads via email to verify our preliminary assessments, which we did via in-person meetings, telephone calls and email interviews. Email reminders were sent to non-respondents. We did this for each phase of the study. This process was given ethical approval by the University of Leeds Faculty of Medicine and Health Research Ethics Committee (MREC 15-009). We obtained written consent from all participants. Interview data were anonymised apart from cases demonstrating examples of good practice. The verification process involved scrutinising the completed checklists for the specific audit feedback reports and the interviewees advising us of any disagreements with our findings. We also asked audit leads whether they used additional methods of feedback to disseminate audit findings and recommendations. The interviews were for confirmatory purposes only.

Over the period of our three content analyses, HQIP continued its usual practice of holding educational events and publishing guidance for audit programme leads. For example, *Reporting for Impact*, published in March 2016 (after our first analysis), suggested a framework for feedback reports and covered issues such as defining target audiences, writing executive summaries and presenting findings in multiple formats [8].

We adhered to STROBE guidance for reporting cohort studies [9].

## Results

We initially identified 26 active audit programmes commissioned by HQIP, but only 23 programmes had feedback reports published on the HQIP website as of November 2015. We reviewed these 23 feedback reports and subsequently reviewed 20 and 14 repeat feedback reports in 2017 and 2019 respectively. The National Cardiovascular Audit Programme brought together six cardiovascular domains into one national clinical audit during 2018 and hence our total number of identified feedback reports fell significantly. Six audit leads participated in verification interviews for the 2015 and 2017 data and seven for 2019. The interviews did not identify any discrepancies from our data extraction.

Feedback reports consistently included a number of strengths at baseline (Table 1), including past or planned repeated audit cycles (21; 91%), stating the importance of the audit topic in relation to patient care (22; 93%), presenting authorship as a trusted source (23; 100%), using multi-modal data presentation (23; 100%) and summarising key findings (23; 100%). However, less than half specifically linked recommendations to audit standards, using behaviourally specific action plans, and highlighting positive feedback when a standard had been achieved or performance had significantly improved.

**Table 1:** Feedback reports from national clinical audit programmes meeting assessment criteria

Domain	Criterion	Number and proportion of feedback reports meeting criterion					
		November 2015 (*n* = 23)		January 2017 (*n* = 20)		August 2019 (*n* = 14)	
**Audit components**	Data are based on recent performance (less than 6 months) [2].	2	9%	1	5%	0	0%
	Audit cycles are repeated or intended to be repeated [2].	21	91%	19	95%	14	100%
	Data are about the individual’s or team’s own behaviour(s), i.e. regional data included [2].	18	78%	16	80%	8	57%
	Importance of audit topic as related to patient care is clearly stated [7].	22	96%	20	100%	14	100%
**Feedback components**	Authorship of the feedback report is identified as a trusted source (e.g. recognised professional body) [2].	23	100%	20	100%	14	100%
	A specific dissemination list is provided for the feedback report [7].	4	17%	18	90%	7	50%
	Presentation is multi-modal including either text and talking or text and graphical materials [2].	23	100%	19	95%	14	100%
	National data are displayed in graphical form [2].	21	91%	18	90%	13	93%
	Regional data are displayed in graphical form [2].	13	57%	10	50%	7	50%
	A short or summarised version of the feedback report is available on the website [7].	1	4%	5	25%	4	29%
	Key audit standards are present [2].	18	78%	18	90%	13	93%
	Key audit standards are easily identified within the document, e.g. highlighted text/bullet points/text box [2].	14	61%	18	90%	12	86%
	Key audit findings are present [7].	23	100%	20	100%	14	100%
	Key audit findings are easily identified within the document, e.g. highlighted text/bullet points/text box [7].	18	78%	20	100%	14	100%
	Audit recommendations are present [1].	18	78%	19	95%	14	100%
	Audit recommendations are easily identified within the document, e.g. highlighted text, bullet points, text box [7].	15	65%	19	95%	14	100%
**Enhanced feedback**	Recommendations are clearly linked to audit standards [1].	6	26%	16	80%	13	93%
	Action plans are phrased in a behaviourally specific manner (who, what, when, where) [7].	9	39%	19	95%	14	100%
	Actions plans are easily identified within the document, e.g. highlighted text, bullet points, text box [1].	9	39%	17	85%	13	93%
	Positive feedback is highlighted when a standard has been achieved or where there is significant improvement since a previous audit [7].	10	43%	9	45%	11	79%
**Feedback includes multiple comparators for national performance**	Audit standards [7].	12	52%	18	90%	13	93%
	Past performance [7].	18	78%	17	85%	14	100%
	Achievable benchmark (e.g. top 10%) [7].	2	9%	8	40%	7	50%
	Regional comparators [7].	11	48%	15	75%	9	64%
**Feedback includes multiple comparators for regional performance**	Audit standards [7].	4	17%	14	70%	7	50%
	Past performance [7].	5	22%	9	45%	0	0%
	Achievable benchmarks (e.g. top 10%) [7].	0	0%	9	45%	2	14%
	Regional comparators [7].	18	78%	15	75%	8	57%
	National average [7].	12	52%	14	70%	8	57%

We observed improvements over our subsequent two assessments, so that by 2019, at least 13 (93%) of sampled audits presented easily identifiable key audit findings and recommendations, linked recommendations to audit standards, and proposed easily identifiable, specific action plans. Whilst the use of regional comparators did not generally improve, audit leads highlighted that programmes were now more likely to provide local comparative data via additional regional reports and real-time data for individual units available online.

The main shortcoming was the time lag between data collection and feedback; none of the 14 reports assessed in 2019 presented performance data less than six months old. Audit leads did highlight that some of these data might be available sooner via programme websites. They also reported a variety of other methods of feedback, including oral presentations, clinician workshops, outlier-focused feedback, patient reports, commissioners and funder reports, email correspondence to clinical leads, online real-time hospital data, quarterly reports and visual slideshows sent to individual units.

We observed continuing scope for improvement in other criteria. For example, half or less of the most recent sample provided a specific dissemination list, a shortened or summary version of the feedback report and achievable benchmarks.

## Discussion

We have observed improving adherence to best practice for published feedback from UK national clinical audit programmes. We have also identified further scope for improving feedback methods which, given the wide reach of national clinical audit programmes, could have significant impacts on health service delivery and patient outcomes if applied more consistently.

To the best of our knowledge, this is the first criterion-based assessment of published feedback reports from national clinical audits. We identified improving trends in feedback reports, including the identification of key audit standards, findings and recommendations, the greater use of summaries, the definition of target groups for dissemination and the presentation and specification of action plans which are also more clearly linked to audit standards.

One key logistical challenge for many national audits concerns how to reduce time lags between data collection and feedback. Only three out of the total of 57 feedback reports we assessed included data collected within the preceding 6 months. As performance data can be published online before the formal report, we presumably over-estimated the gap between data collection and feedback in several cases. Nevertheless, even allowing for wider and faster dissemination channels, this discontinuity in the audit cycle means that recipients may discount performance data they consider outdated [10]. It is important to note that several national clinical audit programmes have created real-time feedback portals online, allowing for individual units and even individual clinicians to review their performance against confidential comparative data. These portals typically present raw data with no accompanying feedback in the form of recommendations or action plans. However, there is scope for enhancing such platforms to both diminish time lags between data collection and feedback whilst applying other evidenced-based feedback methods.

Our evaluation had several limitations. First, we applied all criteria to every audit report, regardless of the history of each audit. For example, a first audit report which did not have any previous feedback data available for comparison was rated as not meeting our criterion of showing comparisons with previous performance. We took this approach to simplify application of our criteria and reduce subjective judgements within the content analyses. In this case, it means that some improvements observed at follow-up may have occurred because performance data became available for repeat audit reports.

Second, we focused only on the most recent feedback report published on the HQIP website. As we highlighted, several audit programmes publish feedback elsewhere, for example, in some cases regional feedback reports are circulated separately. Limiting our analyses to feedback reports published on the HQIP site risked underestimating the extent to which audit programmes as a whole fulfilled our best-practice criteria for feedback. We tried to overcome this limitation by the verification process, although most audit programmes did not respond to the invitation. Whilst our analysis of reports undoubtedly underestimates the range of dissemination activities accompanying national clinical audits, most of our assessment criteria focused on ‘upstream’ features with substantial ‘downstream’ influence, such as the repetition of audit cycles, the clarity of audit standards and recommendations and the inclusion of behaviourally specific action plans.

Third, the reduced number of audit programmes assessed in 2017 and 2019 limited the reliability and generalisability of our findings.

Fourth, whilst our data suggest marked improvements in the content of audit reports, we cannot make any causal inferences about the effects of HQIP activities, such as the *Reporting for Impact* guidance [8]. Furthermore, the HQIP guidance did not include all the best practice recommendations we assessed. A range of other initiatives have been on-going, aimed at evaluating and enhancing the effectiveness of audit. Cochrane reviews have identified features of more effective change and Brehaut et al. have published 15 state-of-the-science, theory-informed recommendations for effective feedback interventions [11]. We recommend that any future assessments of national clinical audit programmes are based upon refined best-practice criteria.

## Conclusion

National audits represent considerable investments in infrastructure as well as the time of staff involved in collecting data locally (where routinely collected data are not available). However, they also have the potential for high reach and impact if feedback channels and content can be optimised. Our work has identified priorities for improvement, but there are still important gaps in the evidence base for audit and feedback. Implementation laboratories involve embedding sequential head-to-head trials testing different enhancements of interventions within an established improvement initiative; the resulting incremental changes accumulate to deliver larger improvement [12]. National clinical audit programmes present opportunities for such experimental work to evaluate varying forms of feedback [13].

Furthermore, a criterion-based assessment of feedback from national clinical audit programmes offers an efficient means of monitoring their quality and guiding improvement. We welcome further work to improve upon our methods, including the refinement of best-practice criteria [11].

## Supplementary Information

**Additional file 1.** Checklist.

**Additional file 2.** Audit description table.

**Additional file 3.** Populated STROBE cohort study checklist.

## Data Availability

All anonymised data generated or analysed during this study are included in this published article. Additional data from the six short interviews could not be sufficiently anonymised because of the small numbers and our consent did not allow for data sharing; therefore, this dataset has restricted access (further information available from corresponding author upon request).

## Abbreviations

HQIP: Healthcare Quality Improvement Partnership
NIHR: National Institute for Health Research
