# Supplementary material for: Repeated analyses of national clinical audit reports demonstrate improvements in feedback methods
Source: Implement Sci Commun. 2020 Nov 25;1:106. doi: 10.1186/s43058-020-00089-3 (PMC7691059; doi:10.1186/s43058-020-00089-3)
Supplement: Supplementary file 1 — Additional file 1. Checklist. [file 43058_2020_89_MOESM1_ESM.docx]

**National Clinical Audit - Report and Feedback Analysis – checklist**

Report Title:

Date published:

Named Audit Manager:

Details of interviewee:

Mode of interview: telephone/email/other……………….

Q.1. Number of pages for full report –

| Q. | TIME-SCALE OF DATA COLLECTION |  | | | | Page Ref: | Comments | Data source: | |
| --- | --- | --- | --- | --- | --- | --- | --- | --- | --- |
| 2. | Date of last data point collection: |  | | | |  |  | Audit Report | Interview |
| 3. | Date of feedback: |  | | | |  |  |  |  |
| 4. | Is feedback based on recent performance? | <3months | 3-6months | 6-12months | >12months |  |  |  |  |

| Q. | AUDIT DETAILS | YES | NO | UNCLEAR | Page  Ref: | Comments | Data Source: | |
| --- | --- | --- | --- | --- | --- | --- | --- | --- |
| 5. | Is this a follow-on audit? |  |  |  |  |  | Audit Report | Interview |
| 6. | Have re-audits been planned? |  |  |  |  |  |  |  |
| 7. | Is there a recommended SPECIFIC dissemination list (target audience) for the audit report? |  |  |  |  |  |  |  |
| 8. | Is the authorship of the audit report identified as a trusted source (recognised professional body)? |  |  |  |  |  |  |  |

| Q. | Levels of Feedback: | | | Summary | Page ref: | Summary with full report | Example Page ref: | Comments | Date source: | |
| --- | --- | --- | --- | --- | --- | --- | --- | --- | --- | --- |
|  |  |  |  |  |  |  |  |  | Audit report | Interview |
| 9. | Present as separate document | | |  |  |  |  |  |  |  |
| 10. | Contains graphical materials | | |  |  |  |  |  |  |  |
| 11. | Importance of audit topic stated as related to patient care | | |  |  |  |  |  |  |  |
| 12. | Key expected behaviour specified | | |  |  |  |  |  |  |  |
| 13. | Key audit standards present (expected benchmark) | | |  |  |  |  |  |  |  |
| 14. | Key expected behaviour/standards easily identified within document  e.g. highlighted text/bullet points/text box | | |  |  |  |  |  |  |  |
| 15. | Key findings present | | |  |  |  |  |  |  |  |
| 16. | Key findings easily identified within document  e.g. highlighted text/bullet points/text box | | |  |  |  |  |  |  |  |
| 17. | Recommendations present | | |  |  |  |  |  |  |  |
| 18. | Recommendations easily identified within document  e.g. highlighted text/bullet points/text box | | |  |  |  |  |  |  |  |
| 19. | Recommendations clearly linked to criteria/standards | | |  |  |  |  |  |  |  |
| 20. | Action Plans (phrased in a behaviourally specific manner - who, what, when, where) present | | |  |  |  |  |  |  |  |
| 21. | Action Plans easily identified within document  e.g. highlighted text/bullet points/text box | | |  |  |  |  |  |  |  |
| 22. | Positive feedback clearly highlighted when a standard has been achieved | | |  |  |  |  |  |  |  |
| 23. | Is current National performance compared against multiple comparators? | | |  |  |  |  |  |  |  |
|  | | | Audit standards |  |  |  |  |  |  |  |
|  | | | Past performance (as part of re-audit) |  |  |  |  |  |  |  |
|  | | | Achievable benchmarks (e.g. top 10%) |  |  |  |  |  |  |  |
|  | | | Regional comparators |  |  |  |  |  |  |  |
| 24. | Is local/site specific performance compared against multiple comparators? | | |  |  |  |  |  |  |  |
|  | | Audit standards | |  |  |  |  |  |  |  |
|  | | Past performance (as part of re-audit) | |  |  |  |  |  |  |  |
|  | | Achievable benchmarks (e.g. top 10%) | |  |  |  |  |  |  |  |
|  | | Regional comparators | |  |  |  |  |  |  |  |
|  | | National average | |  |  |  |  |  |  |  |
| 25. | Is national comparison data in graphical form? | | |  |  |  |  |  |  |  |
| 26. | Is local/site specific comparison data in graphical form? | | |  |  |  |  |  |  |  |

AUDIT REPORT – ADDITIONAL FEEDBACK FROM INTERVIEWS:

| Q.  27 | Apart from report dissemination is audit feedback delivered via other means? | Oral presentation | Email correspondence | Other:  Specify ……………. | Data Source: | |
| --- | --- | --- | --- | --- | --- | --- |
|  |  |  |  |  | Audit Report | Interview |

| Q.  28 | Who are the target audience for this additional feedback? | Hospital Audit leads | NHS staff: speciality specific | NHS staff: non-speciality specific | Patients | Other:  Specify ……….. | Data Source: | |
| --- | --- | --- | --- | --- | --- | --- | --- | --- |
|  |  |  |  |  |  |  | Audit Report | Interview |

| Q.  29 | Are the recipients of the feedback identified as having key roles in implementing the suggested target behaviours? | YES | NO | UNCLEAR | Comments | Data Source: | |
| --- | --- | --- | --- | --- | --- | --- | --- |
|  |  |  |  |  |  | Audit Report | Interview |

| Q.  30 | Is the feedback presentation mandatory for the target audience? | YES | NO | UNCLEAR | Comments | Data Source: | |
| --- | --- | --- | --- | --- | --- | --- | --- |
|  |  |  |  |  |  | Audit Report | Interview |

| Q.  31 | Who is appointed to present the feedback data? | Member of audit team | Hospital Audit lead | Other:  Specify …………….. | Data Source: | |
| --- | --- | --- | --- | --- | --- | --- |
|  |  |  |  |  | Audit Report | Interview |

| Q.  32 | For oral presentations…where did they take place? | Organisation where target audience is based | Target audience asked to attend separate presentation meeting | Other:  Specify ……………. | Data Source: | |
| --- | --- | --- | --- | --- | --- | --- |
|  |  |  |  |  | Audit Report | Interview |

| Q.  33 | Was there any follow-up after the feedback delivery? | YES | NO | UNCLEAR | Comments | Data Source: | |
| --- | --- | --- | --- | --- | --- | --- | --- |
|  |  |  |  |  |  | Audit Report | Interview |
