# Supplementary material for: Repeated analyses of national clinical audit reports demonstrate improvements in feedback methods
Source: Implement Sci Commun. 2020 Nov 25;1:106. doi: 10.1186/s43058-020-00089-3 (PMC7691059; doi:10.1186/s43058-020-00089-3)
Supplement: Supplementary file 2 — Additional file 2. Audit description table. [file 43058_2020_89_MOESM2_ESM.docx]

| **Audit Programme** | **Description** | **Audit Feedback Reports Analysed** | | |
| --- | --- | --- | --- | --- |
|  |  | **November 2015** | **January 2017** | **August 2019** |
| National Bowel Cancer Audit Programme | Established in its current form in 2005 as an audit of surgical practice in bowel cancer patients. Since 2015, the scope of the audit has expanded to include other treatments for bowel cancer in secondary care, as well as evaluating the processes of care and outcomes. The programme is delivered by the Clinical effectiveness Unit at the Royal College of Surgeons of England, NHS Digital and the Association of Coloproctology of Great Britain and Ireland. | Bowel Cancer Audit Report 2014 | National Bowel Cancer Audit Report 2016 | National Bowel Cancer Annual Report 2018 |
| National Oesophago-gastric Cancer Audit Programme | Started in 2006 to evaluate the care of patients with a histological diagnosis of oesophago-gastric cancer in England and Wales. The audit is led by the Association of Upper GI Surgeons, and British Society of Gastroenterology and the Royal College of Radiologists, and supported by the Royal College of Surgeons of England. | National Oesophago-Gastric Cancer Audit 2013 | National Oesophago-Gastric Cancer Audit 2016 | National Oesophago-Gastric Cancer Annual report 2018 |
| National Head and Neck Cancer Audit | Commissioned to facilitate the delivery of head and neck cancer care in England and Wales over 2005-17. Initially delivered by NHS Digital and subsequently by Saving Faces (The Facial Surgery Research Foundation) and is now independent of the National Clinical Audit and Patient Outcomes Programme (NCAPOP). | National Head and Neck Cancer Audit Report 2014 (published 2015) |  |  |
| National Lung Cancer Audit | Started in 2005 to measure the outcomes of patients with lung cancer in the UK and facilitate improvements in key determinants of survival. The audit is being managed by the Royal College of Physicians. | National Lung Cancer Audit Report 2014 – Report for the audit period 2013 | National Lung Cancer Audit Report 2015 | National Lung Cancer Audit Report 2018 – for the audit period 2017  (Published 2019) |
| National Prostrate Cancer Audit | Started in 2013 to collect data on the care of men diagnosed with prostate cancer in England and Wales. The programme is led by the Royal College of Surgeons. | National Prostate Cancer Audit: first year annual report – organisation of services and analysis of existing clinical data  (November 2014) | National Prostate Cancer Audit Report 2016 | National Prostrate Cancer Audit: Annual Report 2018  (Published 2019) |
| The National Chronic Kidney Disease Audit | A 3-year project implemented in 2015 in England and Wales to identify and manage patients with CKD in primary care. The audit was delivered by Informatica working with UCL centre for Nephrology, London School of Hygiene and Tropical Medicine, Clinical Effectiveness Group QMUL, with input from the Patient Liaison Group. | The National Chronic Kidney Disease Audit Pilot Report June-December 2014 (Published 2015) | National Chronic Kidney Disease Audit. National Report (Part 1)  (Published 2017) |  |
| National Asthma and COPD Audit Programme | Started in 2013, initially looking at improving outcomes for patients admitted to secondary care with exacerbations of COPD. The scope was subsequently extended to managing patients in primary care, pulmonary rehabilitation and in 2018 Asthma was added to the audit programme. The programme is managed by the Royal College of Physicians. | National COPD Audit Programme: Clinical audit of COPD exacerbations admitted to acute units in England and Wales 2014  (Published 2015) | National COPD Audit Report: Primary care for 2014-15 (Published 2016) | National COPD Audit Programme: Clinical audit of COPD exacerbations admitted to acute hospitals in England and Wales 2017  (Published 2018) |
| National Audit of Dementia | Established in 2008 to evaluate the care received by patients with Dementia in hospitals in England and Wales. The programme is managed by the Royal College of Psychiatrists. | National Audit of Dementia Care in General Hospitals 2012-13: Second Round Audit Report and Update |  | National Audit of Dementia Care in General Hospitals 2018-2019: Round Four Audit Report |
| National Adult Diabetes Audit Programme | First published in 2005 and is now the largest annual clinical audit in the world, evaluating the effectiveness of diabetes care in England and Wales against the NICE Clinical Guidelines and NICE quality Standards Delivered by NHS Digital in collaboration with Diabetes UK. | National Diabetes Audit 2012-2013 – Report 2: Complications and Mortality  (Published 2015) | National Diabetes Audit 2015-2016 and 2014-2015 – Report 1: Care Processes and Treatment Targets (Published 2017) | National Diabetes Audit, 2017-18 – Report 1: Care Processes and Treatment Targets  (Published 2019) |
| National Paediatric Diabetes Audit | Established to evaluate the care and outcomes for children and young adults with diabetes receiving care from Paediatric Diabetes Units in England and Wales. Delivered by the Royal College of Paediatrics and Child Health since 2010. | National Paediatric Diabetes Audit 2013-14  (Published 2015) | National Paediatric Diabetes Audit 2015-2016  Part 1: Care Processes and Outcomes  (Published 2017) | National Paediatrics Diabetes Audit Report 2017-18: Care Processes and Outcomes  (Published 2019) |
| National Emergency Laparotomy Audit | Established in 2013 to improve the key factors which determine the outcome of Emergency Laparotomy which is a surgery associated with high mortality. The audit is managed by the Royal College of Anaesthetists. | The first patient report of the national emergency laparotomy audit  (Published 2015) | The second patient report of the National Emergency Laparotomy Audit (NELA)  December 2014 to November 2015  (Published 2016) | The Fourth Patient Report of the National Emergency Laparotomy Audit (NELA) December 2016 to November 2017  (Published 2018) |
| Falls and Fragility Fracture Audit Programme (FFFAP) | The FFFAP is run by the Royal College of Physicians and is designed to audit the hospital care of patients with fragility fractures and inpatient falls. Comprised of three components: National Hip Fracture Database; Fracture Liaison Service Database; and National Audit of Inpatient Falls | National audit of inpatient falls, Audit Report 2015 | National Hip Fracture Database – annual report 2016 | National Hip Fracture Database – annual report 2018 |
| National Cardiac Audit Programme | Reports on six major national clinical audits, evaluating the care of patients with heart disease in the UK. The separate audits were amalgamated into one report in 2018, with a focus on patient outcomes, safety and clinical effectiveness | National Audit of Cardiac Ablation 2013-14  (Published 2015) | National Audit of Cardiac Rhythm Management Devices April 2015-March 2016  (Published 2017) | National Cardiac Audit Programme Annual Report 2018 |
|  |  | National Adult Cardiac Surgery Audit – Annual Report 2010-2011  (Published 2012) |  | National Cardiac Audit Programme Annual Report 2018 |
|  |  | National Congenital Heart Disease Audit Report 2011-2014  (Published 2015) | National Congenital Heart Disease Audit Report 2012-15  (Published 2016) | National Cardiac Audit Programme Annual Report 2018 |
|  |  | National Heart Failure Audit, April 2013 – March 2014  (Published 2015) | National Heart Failure Audit, April 2014-March 2015  (Published 2016) | National Cardiac Audit Programme Annual Report 2018 |
|  |  | Myocardial Ischaemia National Audit Project. Annual report April 2013 – March 2014 | Myocardial Ischaemia National Audit Project. Annual report April 2014 – March 2015  (Published 2017) | National Cardiac Audit Programme Annual Report 2018 |
|  |  | National Audit of Percutaneous Coronary Interventional Procedures – Annual Public Report January 2012 – December 2012  (Published 2014) | National Audit of Percutaneous Coronary Interventions – Annual Public Report 2014  (Published 2016) | National Cardiac Audit Programme Annual Report 2018 |
| Inflammatory Bowel Disease National Clinical Audit Project | Commissioned in England and Wales between 2006-2017 to measure the efficacy, safety and appropriate use of biological therapies in patients with IBD. Managed by the Royal College of Physicians. | National Clinical Audit of Biological Therapies: UK inflammatory bowel disease (IBD) audit – Adult report September 2015 | National Clinical Audit of Biological Therapies: UK inflammatory bowel disease (IBD) audit – Annual report September 2016 |  |
| National Neonatal Audit Programme | Established in 2006 to assess care received by babies admitted to neonatal units in England Wales, and facilitate the improvements in quality of care and patient outcomes. The programme is lead by the Royal College of Paediatrics and Child Health. | National Neonatal Audit Programme. Extended online version – Annual Report 2013  (Published 2014) | National Neonatal Audit Programme. 2015 annual report on 2014 data  (Published 2015) | National Neonatal Audit Programme. 2018 annual report on 2017 data  (Published 2018) |
| Paediatric Intensive Care Audit Network (PICANet) | Established in 2002 to facilitate improvements in the provision of paediatric intensive care in the UK. It audits the quality of care delivery against the standards delivered by the Paediatric Intensive Care Society. | PICANet: A Decade of Data – Paediatric Intensive Care Audit Network  (Published 2014) | PICANet: November 2016 Annual Report | PICANet: Annual Report 2018  Summary Report Data collection period January 2015-December 2017  (Published 2018) |
| National Joint Registry (NJR) | The registry was set up in 2002 by the Department of Health and Welsh Government to collect information on joint replacement operations. Submission for NHS organisations has been mandatory since 2011. It is the largest registry of its kind in the world, holding over 3 million records on joint replacement operations, with the aim to monitor performance and improve outcomes. | National Joint Registry for England, Wales and Northern Ireland, 11^th^ Annual Report, 2014 | National Joint Registry for England, Wales and Northern Ireland, 13^th^ Annual Report, 2016 |  |
| Sentinel Stroke National Audit Programme (SSNAP) | The audit evaluates the quality of the organisation and delivery of inpatient care in England, Wales and Northern Ireland for patients admitted following a stroke. In 2010 the audit changed from a retrospective audit of hospital case notes to a prospective audit using a real-time webtool. | How good is stroke care? First SSNAP Annual Report. Care received from April 2013 to March 2014 (Sentinel Stroke National Audit Programme) | SSNAP acute organisational audit report (Published 2016) | SSNAP Clinical Audit April 2013 – March 2018  Annual Public Report  (Published 2019) |
